# Hydrogen-rich saline reduces cell death through inhibition of DNA oxidative stress and overactivation of poly (ADP-ribose) polymerase-1 in retinal ischemia-reperfusion injury

**DOI:** 10.3892/mmr.2015.3731

**Published:** 2015-05-05

**Authors:** HONGWEI LIU, NING HUA, KELIANG XIE, TINGTING ZHAO, YONGHAO YU

**Affiliations:** 1 Department of Anesthesiology, Tianjin Institute of Anesthesiology, Tianjin Medical University General Hospital, Tianjin 300052, P.R. China; 2 Department of Pediatric Ophthalmology and Strabismus, Tianjin Medical University Eye Hospital, Tianjin 300052, P.R. China

**Keywords:** oxidative stress, retinal ischemia-reperfusion injury, poly (ADP-ribose) polymerase-1, hydrogen-rich saline

## Abstract

Overactivation of poly (ADP-ribose) polymerase 1 (PARP-1), as a result of sustained DNA oxidation in ischemia-reperfusion injury, triggers programmed cell necrosis and apoptosis. The present study was conducted to demonstrate whether hydrogen-rich saline (HRS) has a neuroprotective effect on retinal ischemia reperfusion (RIR) injury through inhibition of PARP-1 activation. RIR was induced by transient elevation of intraocular pressure in rats. HRS (5 ml/kg) was administered peritoneally every day from the beginning of reperfusion in RIR rats until the rats were sacrificed. Retinal damage and cell death was determined using hematoxylin and eosin and terminal deoxynucleotidyl transferase dUTP nick end labeling staining. DNA oxidative stress was evaluated by immunofluorescence staining of 8-hydroxy-2-deoxyguanosine. In addition, the expression of PARP-1 and caspase-3 was investigated by western blot analysis and/or immunohistochemical staining. The results demonstrated that HRS administration improved morphological alterations and reduced apoptosis following RIR injury. Furthermore, the present study found that HRS alleviated DNA oxidation and PARP-1 overactivation in RIR rats. HRS can protect RIR injury by inhibition of PARP-1, which may be involved in DNA oxidative stress and caspase-3-mediated apoptosis.

## Introduction

Retinal ischemia reperfusion (RIR) injury exists in various eye diseases, including glaucoma, diabetic retinopathy and other ocular vascular disorders (1–3). Production of reactive oxygen species (ROS) in RIR induces oxidative stress, which leads to lipid oxidation, protein synthetic disorder and DNA oxidation (4,5). It has been verified that nuclear and mitochondrial DNA oxidation leads to neuron death (6–8). Therefore, the prevention of DNA injury may be an effective strategy to promote retinal cellular survival.

ROS, particularly peroxynitrite, can induce nuclear DNA oxidative breaks, followed by activation of poly (ADP-ribose) polymerase-1 (PARP-1), a nuclear enzyme involved in the regulation of multiple pathophysiological cellular procedures, including DNA repair, gene transcription and cell death (9–11). PARP-1 catalyzes the formation of poly (ADP-ribose) polymers, which triggers the translocation of apoptosis-inducing factor (AIF) from the mitochondria into the nucleus, causing DNA condensation and caspase-independent cell death. In addition, ROS induced mitochondrial DNA stress and lipid oxidation and can also induce mitochondrial structural breakdown and the release of cytochrome *c*, which promotes caspase family activation and apoptotic cell death (8,12).

Hydrogen, the most common gas in the atmosphere, was initially recognized as a therapeutic reductive substance in medicine in 1975 (13). Several studies (14–17) regarding its usage and underlying molecular mechanism have been conducted since Ohsawa *et al* identified that the inhalation of hydrogen gas markedly suppressed ischemia-reperfusion injury in the brain by alleviating oxidative stress caused by ROS in 2007 (18). Hydrogen can be dissolved in water up to 0.8 mM under atmospheric pressure at room temperature and its solubilized form, hydrogen-rich saline (HRS), is beneficial since it is a portable, easily administered and a safe means of delivering hydrogen (14). In addition, administration of HRS has been demonstrated to enhance cell survival in different disorders in animal models, particularly due to its anti-oxidative and anti-inflammatory properties (15–17).

The present study aimed to investigate whether HRS had a protective effect in rodent RIR injury. In addition, the mechanism underlying the protective effects of HRS was investigated by measuring DNA oxidative stress, PARP-1 expression and apoptosis.

## Materials and methods

### Animals

A total of 48 3 month-old adult male Sprague-Dawley rats weighing 300–350 g (Tianjin Medical University Animal Center, Tianjin, China) were used in the present study. Animals were fed a standard rodent diet with a normal light-dark cycle. All animals were cared for with the approval of the Laboratory Animal Research Committee at Tianjin Medical University Eye Center. All experimental procedures involving animals were performed in accordance with the ARVO Statement for the Use of Animals in Ophthalmic and Vision Research.

### HRS production

Hydrogen was dissolved and saturated in 0.9% normal saline (NS) for 6 h under a high pressure of 0.4 MPa using hydrogen-producing apparatus (GCH-3000; Tianjin Tongpu Analytical Instrument Tech Co., Ltd., Tianjin, China) according to the method reported by Cai *et al* (17). The saturated HRS was stored at 4°C under normal pressure in an aluminum bag. HRS was freshly prepared and sterilized by γ radiation 24 h prior to the experiment, ensuring an effective concentration of 0.6 mM. The content of hydrogen in NS was confirmed through gas chromatography as described previously by Ohsawa *et al* (14).

### Rat RIR model and peritoneal administration of HRS

All surgery was performed under general anesthesia with intraperitoneal administration of chloral hydrate (300 mg/kg). The anterior chamber of the left eye was cannulated with a 30-gauge needle connected to a reservoir containing NS. Intraocular pressure was increased to 130 mmHg for 60 min and ocular ischemia was confirmed by interruption of the ocular circulation, as described by Sun *et al* (2). Thereafter, the cannula was immediately retracted and the adequacy of retinal reperfusion was confirmed visually by ophthalmoscopy. Rats that did not recover from retinal perfusion 3 min after the end of the ischemic period and those with lens injury, which prevents retinal ganglion cell (RGC) death and promotes axonal regeneration (19), were excluded from the investigation. Following surgery, levofloxacin ophthalmic solution 0.3% (Cravit^®^; Santen Pharmaceutical Co., Ltd., Osaka, Japan) was dropped into the conjunctival sac of the left eye to prevent infection. The model of IR injury was fully induced 24 h after reperfusion.

All the rats were classified into the following four groups: IR injury with HRS group, IR injury with NS group, HRS-treated control group and NS-treated control group. The animals in the IR injury with HRS and NS groups were peritoneally injected with HRS or NS (5 ml/g) at the beginning of reperfusion. Consecutive HRS or NS treatment was administered daily until the rats were sacrificed with overdose anaesthesia. The rats in the HRS-treated and NS-treated control groups were peritoneally injected with HRS or NS (5 ml/kg) at the same time points without initial reperfusion. As molecular hydrogen can easily diffuse into tissues and penetrate the cellular membrane, HRS (5 ml/kg) was injected peritoneally only at the beginning of reperfusion. Furthermore, consecutive HRS, compared with the equivalent quantity of NS, was administered daily until the rats were sacrificed.

### Histological staining

Freshly enucleated eyeballs (n=6) were fixed in 4% paraformaldehyde in 0.1 M phosphate-buffered saline (PBS) for 30 min. Subsequently, corneal paracentesis was performed using a 25 G needle. Following that, the entire eye cups were further fixed overnight and then processed for paraffin embedding. Sections (4 *µ*m) were cut along the vertical meridian of the eye, 2 mm away from the optic nerve head and mounted on pre-coated glass slides. Following deparaffinization and rehydration, sections were stained with hematoxylin and eosin. Retinal damage was assessed by measuring the thickness of the retina and cell loss in the ganglion cell layer (GCL). The retinal thickness is defined as the total width between the inner limiting membrane to the interface of the outer plexiform layer and the outer nuclear layer (20). The number of cells in the GCL was calculated under a light microscope (magnification, x40) (BX51; Olympus Corporation, Tokyo, Japan). Three different sections were randomly selected and each section was measured at four different points between 1 and 2 mm from the optic disc. The average value was appointed as the thickness of retina and the number of cells in the GCL of the eye.

### Terminal deoxynucleotidyl transferase dUTP nick end labeling (TUNEL)

Cryosections (n=6) were fixed with 4% paraformaldehyde in 0.1 M PBS (pH 7.4) for 20 min. Cell death was detected by a TUNEL assay using the *In Situ* Cell Death Detection kit, POD (Roche Diagnostics Deutschland GmbH, Mannheim, Germany) and color-developed with 3,3′-diaminobenzidine (DAB) substrate (Roche Diagnostics Deutschland GmbH) according to the manufacturer’s instructions. The number of TUNEL-positive cells in the GCL was counted at x400 magnification for each section using a light microscope (BX51; Olympus Corporation).

### Immunofluorescence staining of 8-hydroxy-2-deoxyguano-sine (8-OHdG)

Eyes (n=6) in optimal cutting temperature compound were cut into 6 *µ*m sections (RM2165; Leica, Wetzlar, Germany) following being flash frozen in liquid nitrogen. The cryosections were fixed in ice-cold acetone for 10 min, washed in PBS three times and blocked with 1% bovine serum albumin (BSA) in PBST for 30 min at room temperature. Subsequently, primary goat anti-8-hydroxyguanine (8-OHdG, a classical DNA oxidative product) polyclonal antibody (1:100; cat. no. ab10802; Abcam, San Francisco, CA, USA) in 1% BSA in PBST were added to the samples and incubated in a humidified chamber overnight at 4°C. Following three washes in PBS, the sections were incubated with tetramethyl rhodamine isothiocyanate-conjugated rabbit anti-goat IgG secondary antibody (1:200; cat. no. BA1091; Beijing Zhongshan Golden Bridge Biotechnology Co., Ltd., Beijing, China) for 1 h in the dark at 37°C, followed by 4′,6-diamidino-2-phenylindole staining (0.1 *µ*g/ml; Sigma-Aldrich, St. Louis, MO, USA) and another three washes in PBS. Coverslides were mounted and the immunoreactivity of 8-OHdG was detected under a fluorescence microscope (DFC500; Leica Microsystems, Renens, Switzerland).

### Western blot analysis

Total retinal tissue lysates (n=6) were prepared by the addition of 500 *µ*l sodium dodecyl sulfate (SDS) buffer [250 mM Tris (pH 6.8), 10% SDS, 500 mM dithiothreitol, 50% glycerol, 0.5% bromophenol blue and a 1:100 protease inhibitor cocktail (cat. no. P8340; Sigma-Aldrich)]. Total tissue extracts (20 *µ*g) were separated on a 12% SDS polyacrylamide gel. Proteins were transferred onto an Immobilon-P membrane (Millipore, Billerica, MA, USA) and blocked with 5% skim milk in TBST (TBS containing 0.05% Tween-20) at room temperature for 2 h. Subsequently, the membranes were hybridized at 4°C overnight in TBST with the primary antibodies: Anti-rPARP-1 (1:5,000; cat. no. 1051-1; Epitomics, Burlingame, CA, USA), and anti-rCaspase-3 (1:1,000; cat. no. 9661; Cell Signaling Technology, Boston, MA, USA) overnight at 4°C. Following incubation with a horseradish peroxidase-conjugated anti-rabbit immunoglobulin antibody, proteins were visualized with an enhanced chemiluminescence kit (Kirkegaard & Perry Laboratories, Inc., Gaithersburg, MD, USA). For normalization, blots were probed with β-actin, a housekeeping antibody (anti-β-actin; 1:1,000; Beijing Zhongshan Golden Bridge Biotechnology Co., Ltd.). The signal was detected by exposing X-ray films to the processed blots and analyzed by laser scanning densitometry (Personal Densitometer; GE Healthcare, Piscataway, NJ, USA).

### Immunohistochemical staining of PARP-1

Paraffin-embedded sections (n=6, 4 *µ*m thick) were deparaffinized and rehydrated. Antigen retrieval was achieved by boiling in 10 mmol/l sodium citrate buffer for 10 min and then steadily cooling to room temperature. Subsequently, the sections were blocked using 3.0% H_2_O_2_ in methanol for 15 min in order to inhibit endogenous peroxidase activity. Following washing in PBS, the sections were incubated overnight at 4°C with monoclonal rabbit anti-PARP-1 primary antibody (cat. no. 1051-1) at a dilution of 1:40 (Epitomics). The sections were incubated with peroxidase-conjugated goat anti-rabbit IgG (Beijing Zhongshan Goldenbridge Biotechnology Co., Ltd.) at the same dilution of 1:500 for 2 h at 37°C. The sections were washed in PBS, developed in prepared DAB chromogen solution, lightly counterstained with hematoxylin, dehydrated and mounted.

### Statistical analysis

All data are presented as the mean ± standard deviation. Data were analyzed using one-way analysis of variance conducted between groups with post hoc analysis through Bonferroni multiple comparisons test. Student’s t-test was used to compare data between two groups. Statistical analyses were conducted using SPSS version 18.0 (SPSS Inc., Chicago, IL, USA). P<0.05 was considered to indicate a statistically significant difference.

## Results

### HRS administration reduces histological disorder following RIR injury

**Figure 1:**
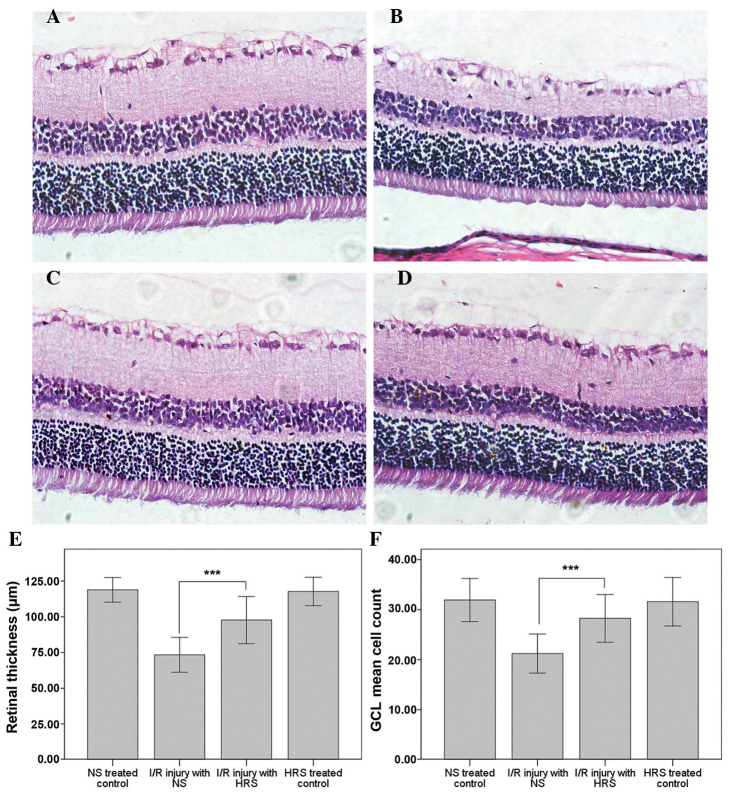
HRS improves histological impairment in a rat model of RIR (magnification, x400). The retinas were sliced after paraffin embedding and stained with hematoxylin and eosin 1 week after RIR injury. (A) Images of representative sections from the NS-treated control group, (B) RIR injury with NS group, (C) the HRS-treated control group and (D) the RIR injury with HRS group are shown. (E) Retinal thicknesses following peritoneal administration of HRS. (F) Number of cells in the GCL following peritoneal administration of HRS. ^***^P<0.0001, compared with I/R-injured retina treated with NS. Histograms represent the mean ±standard deviation. HRS, hydrogen-rich saline; RIR, retinal ischemia reperfusion; GCL, ganglion cells layer; NS, normal saline; I/R, ischemia/reperfusion.

The histological alterations of RIR injury occurred after 1 week of reperfusion (21). The thinning of retinas and cell loss in the GCL demonstrated the successful induction of this model. Abdominal administration of HRS effectively reversed the morphological alterations, including thinning of retinas and cell loss in the GCL (Fig. 1). By contrast, the retinal thickness of the RIR injury with NS group was 73.39±6.07 *µ*m, which was markedly thinner than the retinal thickness in the NS-treated control group (118.85±4.31 *µ*m; P<0.001). The retinal thickness in the HRS-treated control group was 117.71±4.95 *µ*m, which demonstrated that hydrogen did not alter the thickness of the retina in normal rats. However, following HRS administration the thinning of retina following RIR injury partially recovered to 97.72±8.25 *µ*m, which was significantly different to the NS-treated control (P<0.001).

Furthermore, the number of cells in the GCL in the IR injury with NS group decreased to 21.2±1.9, compared with 31.9±2.2 in the NS-treated control (P<0.001). No significant difference was identified between the HRS-treated (31.6±2.4) or NS-treated controls. By contrast, the number of cells in the IR injury with HRS group was 28.3±2.4 (P<0.001).

### HRS administration alleviates death of RGCs in RIR injury

**Figure 2:**
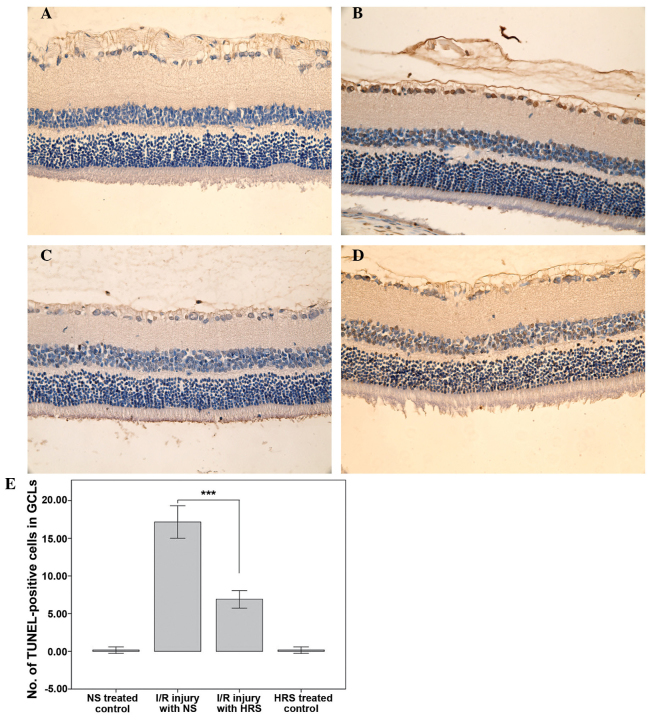
HRS alleviates cell death in the GCL in a rat model of RIR (magnification, x400). Retinal cryosections (n=6) were stained with TUNEL using the *in situ* cell death detection kit 1 week after RIR injury and cell death was detected as brown nuclei by 3,3′-diaminobenzidine staining. (A) Images of representative sections of the NS-treated control group, (B) RIR injury with NS group, (C) the HRS-treated control group and (D) the RIR injury with HRS group are shown. (E) The administration of HRS significantly alleviated cell death in the GCL. ^***^P<0.0001, compared with I/R-injured retina treated with NS. Histograms represent the mean ± standard deviation. HRS, hydrogen-rich saline; RIR, retinal ischemia reperfusion; TUNEL, terminal deoxynucleotidyl transferase dUTP nick end labeling; GCL, ganglion cell layer; NS, normal saline; I/R, ischemia/reperfusion.

The NS-treated control and HRS-treated control had few TUNEL positive cells, which illustrated that HRS did not induce cell death. By contrast, retinas with RIR injury experienced severe cell death in the GCL 7 days after reperfusion, which was in accordance with previous studies (22,23). Treatment with HRS significantly decreased the number of TUNEL-positive cells in the GCL of RIR rats on the seventh day after reperfusion (P<0.001), suggesting that HRS may have a protective effect on cell apoptosis (Fig. 2).

### HRS administration alleviates DNA oxidative breaks in RIR injury

**Figure 3:**
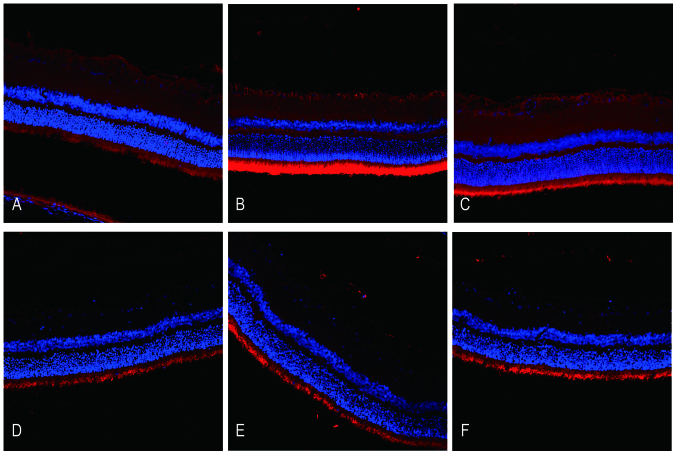
DNA oxidative injury detected by immunoreactivity of 8-OHdG is ameliorated following HRS intervention between 24 h and 7 days after reperfusion in a rat RIR model (magnification, x200). Retinal cryosections (n=6) were stained with primary goat anti 8-OHdG polyclonal antibody (1:100) overnight at 4°C and were incubated with tetramethyl rhodamine isothiocyanate-conjugated rabbit IgG secondary antibody (1:200 for 1 h in the dark at 37°C, followed by 4′,6-diamidino-2-phenylindole staining; 0.1 *µ*g/ml). The immunoreactivity of 8-OHdG was detected under a fluorescence microscope. (A) NS-treated control group, (B) RIR injury with NS group 24 h after reperfusion, (C) RIR injury with NS group 7 days after reperfusion, (D) HRS-treated control group, (E) RIR injury with HRS group 24 h after reperfusion and (F) the RIR injury with HRS group 7 days after reperfusion. HRS, hydrogen-rich saline; 8OHdg, 8-hydroxy-2-deoxyguanosine; RIR, retinal ischemia reperfusion; NS, normal saline.

The DNA oxidative breaks in RIR injury were detected through immunofluorescent staining of 8-OHdG, a DNA oxidative product (Fig. 3). There was moderate 8-OHdG immunoreactivity in the photoreceptor cell layer compared with faint immunostaining in the GCL in the untreated control. Following administration of HRS, 8-OHdG immunoreactivity partially decreased. In addition, IR injury increased the strength of 8-OHdG immunoreactivity in the GCL as well as in photoreceptor cell layers, suggesting severe DNA oxidative stress due to ROS. By contrast, the immunoreactivity of 8-OHdG decreased significantly in the GCL immediately following HRS intervention in IR injury, which may confirm the reductive potential of hydrogen to DNA oxidative stress.

### PARP-1 and cleaved PARP-1 are downregulated following hydrogen intervention, concordant with the expression of caspase-3

Excessive DNA breaks may induce overactivation of PARP-1 and cell death. In addition, as the substrate of caspase-3, activated PARP-1 can be cleaved into 29 and 84 kDa fragments. The primary anti-capase-3 antibody could react with the whole molecule and the 19 kDa fragment of caspase-3, while anti-PARP-1 antibody recognized full length PARP-1 and its 29 kDa fragment.

**Figure 4:**
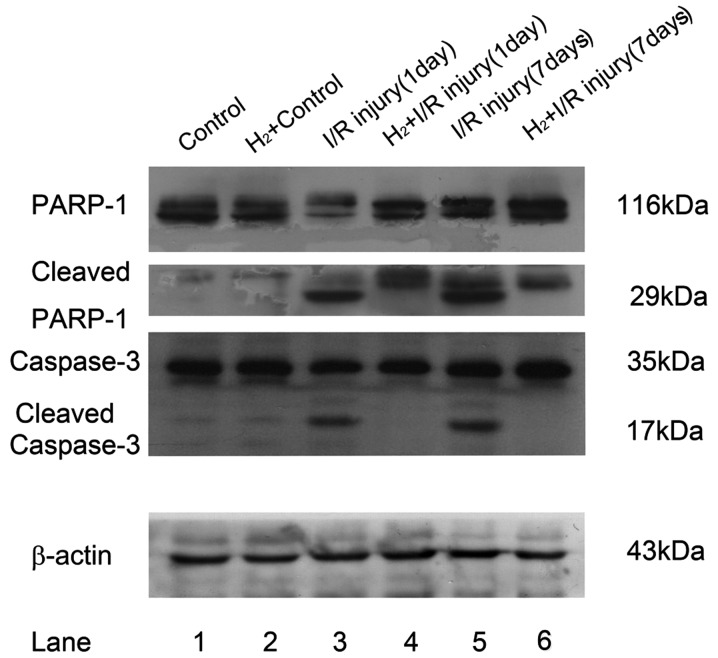
Alterations in PARP-1 and caspase-3 cleavage following administration of HRS in a retinal I/R model in rats. Retinas (n=6) were lysed with RIPA mixed with a protease inhibitor cocktail. Lysates (20 *µ*g) from each group were added into their corresponding layers of 12% SDS-PAGE. Following being transferred onto a polyvinylidene fluoride membrane, primary antibodies (anti-rPARP-1; 1:5,000; anti-rCaspase-3, 1:1,000) were cultured at 4°C overnight followed by horseradish peroxidase-conjugated secondary antibody cultured for 2 h at 37°C. Subsequently, electrochemiluminescnce-assisted exposure was performed to gain 116 kDa full PARP-1 and its cleaved 29 kDa fragments, the intact caspase-3 (35 kDa) and its 17 kDa cleaved fragment. For normalization, blots were probed with β-actin (anti-β-actin; 1:1,000). Compared with the control, RIR injury led to significant cleavage of full length PARP-1 between 24 h and 7 days after reperfusion (lane 1 and 2 versus lane 3 and 5). The cleavage of caspase-3 had a similar change to PARP-1. HRS intervention of RIR injury significantly reduced the cleavage of these two proteins (lane 3 and 5 versus lane 4 and 6). PARP-1, poly (ADP-ribose) polymerase 1; HRS, hydrogen-rich saline; I/R, ischemia/reperfusion.

Cleavage of PARP-1 was significantly elevated in RIR retina 24 h and 7 days after reperfusion (Fig. 4). Compared with the relatively low immunofluorescent staining of PARP-1 at 116 kDa in the untreated control and hydrogen control, the RIR model demonstrated strong immunoreactivity of the 29 kDa C-terminal catalytic domain. At the same time, the pro-form of PARP-1 decreased 24 h after RIR injury, but increased and surpassed the control 7 days after RIR injury, while the 29 kDa cleaved fragment remained visible. Following peritoneal administration of HRS, cleaved PARP-1 was reduced 24 h and 7 days after reperfusion, demonstrated as decreased immunoreactivity of 29 kDa PARP-1 fragments.

The expression of capase-3 was detected in RIR injury by immunostaining. In the untreated control retina and hydrogen control retina, there was almost no caspase-3 immunoreactivity. However, cleaved caspase-3 was detected between 24 h and 7 days after reperfusion. However, hydrogen intervention inhibited caspase-3 cleavage, showing the loss of cleaved fragment immunoreactivity 24 h and 7 days after reperfusion.

### Immunohistochemistry staining of PARP-1 in retina

**Figure 5:**
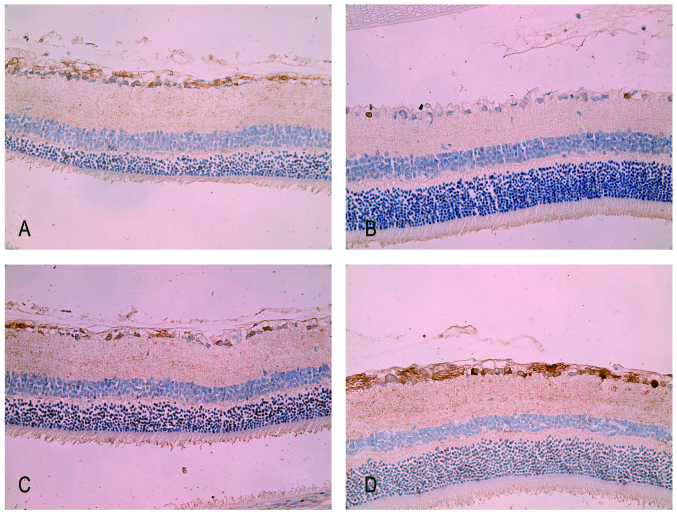
HRS affects the expression and sub-cellular distribution of PARP-1 in the ganglion cell layer in a rat RIR model (magnification, x400). The retinas were sliced after paraffin embedding and stained with anti-rPARP-1 antibody (1:40) and anti-peroxidase-conjugated goat anti-rabbit IgG (1:500) 1 week after RIR injury. The sections were color-developed in 3,3′-diaminobenzidine and lightly counterstained with hematoxylin. (A) Images of representative sections from the NS-treated control group, (B) RIR injury with NS group, (C) HRS-treated control group and (D) RIR injury with HRS group are shown. The immunoreactivity of PARP-1 was concentrated in nuclei of the ganglion cell layer in the RIR group. However, it distributed extensively in the nerve fiber layer and ganglion cell layer following administration of HRS. HRS, hydrogen-rich saline; PARP-1, poly (ADP-ribose) polymerase 1; RIR, retinal ischemia reperfusion.

In normal adult retina, PARP-1 was mildly detected in the cytoplasm of the GCL and nerve fiber layer (Fig. 5A). There were markedly fewer PARP-1 positive cells in the IR injury groups at 7 days. However, the immunoreactivity of PARP-1 was concentrated predominantly in the nuclei of the GCL in IR retina (Fig. 5C). This altered location of PARP-1 from the cytoplasm to the nuclei was previously observed by Huang *et al* (24) in the same rodent experiment model. Since the cleaved 29 kDa fragment of PARP-1 can translocate to the nuclei and bind to the oxidized DNA breaks, the translocation of PARP-1 immunoreactivity illustrated the sustained existence of DNA breaks in RIR injury. By contrast, after 7 days of intraperitoneal administration of HRS in IR rats, there was apparent immunoreactivity of PARP-1 not only in the nuclei but also in the cytoplasm of the GCL as well as in the nerve fiber layer (Fig. 5D).

## Discussion

DNA damage due to oxidative stress is a crucial factor in the pathogenesis of neurodegenerative diseases (25). It has been demonstrated that DNA oxidation is involved in the pathophysiology of retinal disorder (4,26), including RIR injury, which typically manifests as ganglion cell degeneration. Peroxynitrite, generated from NO and O_2−_, destroys lipid, protein and nucleic acid and is considered to be the most effective promoter of cell death (27,28). Since peroxynitrite induces the selective oxidation of guanine in DNA, its oxidative product, 8-OHdG, is the most widely used ‘marker’ for oxidative DNA damage (29–32).

Activation of PARP-1 is recognized to perform central roles in DNA repair, as its DNA binding domain may seal the breaks on DNA strands and prevent incorrect DNA recombination (33). However, severely sustained DNA injury induces overactivation of PARP-1, which in turn leads to increased PAR production that augments the translocation of AIF into the nuclei to induce DNA cleavage into large fragments. This process is known as PARP-1-mediated cell death (PARthanatos) (34–37). Furthermore, the overproduction of PAR can cause lethal consumption of cellular NAD^+^, consequently leading to energy exhausted cell death (37).

In addition, oxidative stress can also cause mitochondria and endoplasmic reticulum to trigger cell apoptosis. For instance, oxidative stress leads to the opening of mitochondrial membrane permeability transition pores and the release of cytochrome *c*, followed by the activation of caspase-3 in the form of cleaved fragments. Since activated caspase-3 has the potential to cleave PARP-1, cell death may be partially inhibited due to its inhibition of PARP-1 activity. Simultaneously, cleaved caspase-3 induces apoptosis, which may be cellular compensation for the severe destruction caused by PARthanatos (38,39). Therefore, PARP-1 and caspase-3 may be simultaneously involved in the procedure and affect each other mutually.

The powerful reductive potential of hydrogen in medicine has been recently recognized (14,16–18). It selectively neutralizes peroxynitrite and hydroxyl radicals generated from oxidative stress and promotes cell survival *in vitro* and *in vivo* (14,40,41). Furthermore, HRS is more conveniently used *in vivo*. The present study demonstrated that peritoneal administration of HRS successfully promoted cell survival in the GCL in a rat RIR model. This result was in accordance with the results of the study by Oharazawa *et al* in which hydrogen loaded eyedrops reduced TUNEL positive cells in nuclear layers of an RIR model (42).

DNA oxidation was significantly reduced following administration of HRS, demonstrating that molecular hydrogen successfully rescued cells by preventing DNA oxidative breaks. Further investigation of the expression of PARP-1 fragments demonstrated its elevated cleavage from the caspase family following IR injury, which was further confirmed by the condensed nuclear aggregation of intact PARP-1 and its 29 kDa cleaved fragment in the GCL in rodent RIR. This result illustrates the overactivation of PARP-1 can cause cell death in RIR. Notably, following treatment with hydrogen, the integrity of PARP-1 was significantly well preserved, suggesting the recovery of its repairing function to maintain stability of neurons and endothelia in retinas.

In addition, caspase-3 cleavage was significantly elevated following IR injury whereas hydrogen intervention reduced caspase-3 cleavage, suggesting that caspase-mediated apoptosis is inhibited by molecular hydrogen.

In terms of oxidative injury in retina, there are several studies which support that PARP-1-mediated cell death may be an important mechanism of RIR injury. Li *et al* (43) found that hydrogen peroxide, a strong oxidizer, induced RGC-5 cell death, which induced abundant production of ROS. The authors found that there was no activation of caspase-3 following oxidative injury, however, PARP and its downstream effector, AIF, were involved (43). Furthermore, Li *et al* (44) investigated D-galactose-induced oxidative stress in neuroblastoma cells and observed significant necrotic cell death, which could not be alleviated by the caspase inhibitor z-VAD-fmk, suggesting that caspase-dependent cell death did not occur. It is possible that PARP-1-mediated cell death co-exists with caspase-dependent apoptosis in RIR injury and molecular hydrogen can modulate these two procedure simultaneously in neuroretina.

Considering the data presented, hydrogen-mediated neuroprotection in the rat retina may result from inhibition of PARP-1. The histological findings strengthen these results, providing morphological evidence of RGC survival. However, further studies on apoptosis and PARthanatos are required in order to confirm the mechanism of hydrogen intervention.
